# The terroir of the finch: How spatial and temporal variation shapes phenotypic traits in DARWIN'S finches

**DOI:** 10.1002/ece3.9399

**Published:** 2022-10-05

**Authors:** Paola L. Carrión, Joost A. M. Raeymaekers, Luis Fernando De León, Jaime A. Chaves, Diana M. T. Sharpe, Sarah K. Huber, Anthony Herrel, Bieke Vanhooydonck, Kiyoko M. Gotanda, Jennifer A. H. Koop, Sarah A. Knutie, Dale H. Clayton, Jeffrey Podos, Andrew P. Hendry

**Affiliations:** ^1^ Redpath Museum, Department of Biology McGill University Montréal Québec Canada; ^2^ Faculty of Biosciences and Aquaculture Nord University Bodø Norway; ^3^ Department of Biology University of Massachusetts Boston Boston Massachusetts USA; ^4^ Centro de Biodiversidad y Descubrimiento de Drogas Instituto de Investigaciones Científicas y Servicios de Alta Tecnología (INDICASAT‐AIP) Panamá República de Panamá; ^5^ Smithsonian Tropical Research Institute Panamá República de Panamá; ^6^ Department of Biology San Francisco State University San Francisco California USA; ^7^ Colegio de Ciencias Biológicas y Ambientales Universidad San Francisco de Quito Quito Ecuador; ^8^ Worcester State University Worcester Massachusetts USA; ^9^ Virginia Institute of Marine Science College of William & Mary Gloucester Point Virginia USA; ^10^ Muséum National d'Histoire Naturelle Département Adaptations du Vivant Bâtiment d'Anatomie Comparée Paris France; ^11^ Department of Biology University of Antwerp Antwerpen Belgium; ^12^ Department of Biological Sciences Brock University St. Catharines Ontario Canada; ^13^ Departement de Biologie Universite de Sherbrooke Quebec Canada; ^14^ Department of Biological Sciences Northern Illinois University DeKalb Illinois USA; ^15^ Department of Ecology and Evolutionary Biology University of Connecticut Storrs Connecticut USA; ^16^ Institute for Systems Genomics University of Connecticut Storrs Connecticut USA; ^17^ School of Biological Sciences University of Utah Salt Lake City Utah USA; ^18^ Department of Biology University of Massachusetts Amherst Amherst Massachusetts USA

**Keywords:** adaptation, adaptive divergence, adaptive radiation, biological diversity, Galápagos landbirds

## Abstract

The term *terroir* is used in viticulture to emphasize how the biotic and abiotic characteristics of a local site influence grape physiology and thus the properties of wine. In ecology and evolution, such terroir (i.e., the effect of space or “site”) is expected to play an important role in shaping phenotypic traits. Just how important is the pure spatial effect of terroir (e.g., differences between sites that persist across years) in comparison to temporal variation (e.g., differences between years that persist across sites), and the interaction between space and time (e.g., differences between sites change across years)? We answer this question by analyzing beak and body traits of 4388 medium ground finches (*Geospiza fortis*) collected across 10 years at three locations in Galápagos. Analyses of variance indicated that phenotypic variation was mostly explained by site for beak size (*η* ^2^ = 0.42) and body size (*η* ^2^ = 0.43), with a smaller contribution for beak shape (*η* ^2^ = 0.05) and body shape (*η* ^2^ = 0.12), but still higher compared to year and site‐by‐year effects. As such, the effect of terroir seems to be very strong in Darwin's finches, notwithstanding the oft‐emphasized interannual variation. However, these results changed dramatically when we excluded data from Daphne Major, indicating that the strong effect of terroir was mostly driven by that particular population. These phenotypic results were largely paralleled in analyses of environmental variables (rainfall and vegetation indices) expected to shape terroir in this system. These findings affirm the evolutionary importance of terroir, while also revealing its dependence on other factors, such as geographical isolation.

## Graphical abstract

The term terroir is used in viticulture to emphasize how the biotic and abiotic characteristics of a local site influence grape physiology and the properties of wine. In ecology and evolution, such terroir (i.e., the effect of space or “site”) is expected to play an important role in shaping phenotypic traits, but how important is the pure spatial effect of terroir in comparison to temporal variation, and the interaction between space and time? We answer this question by analyzing beak and body traits of 4388 medium ground finches (Geospiza fortis) collected across 10 years at three locations in the Galápagos Islands.

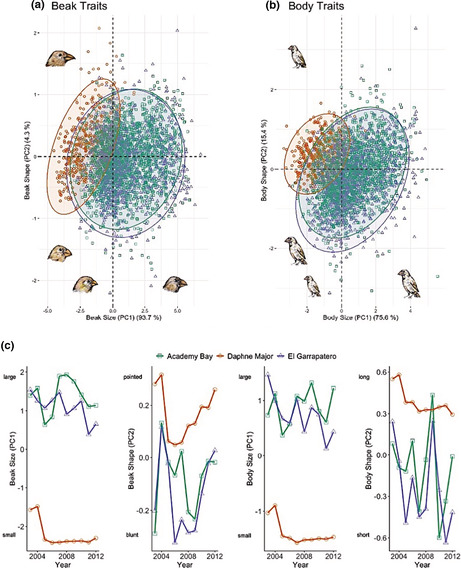

## 1 INTRODUCTION

*Terroir* is considered critical to the properties of wine (Gladstones, 2011; Tonietto & Carbonneau, 2004; Van Leeuwen et al., 2004). Particular combinations of regional and local conditions—both abiotic (elevation, sun exposure, aspect, soil granularity, etc.) and biotic (competitors, predators, parasites, etc.)—strongly shape the physiology of grape vines. Those physiological responses then alter the chemical properties of grapes, which are then detectable in wine. As a result, terroir factors into decisions about which wine varietals (e.g., Pinot Noir or Cabernet Sauvignon) are grown in a given area, in a given vineyard, and in a given “block” (Jones, 2018; Schmidtke et al., 2020). Then, for a given set of these choices, terroir can further influence the color, aroma, and flavor of the resulting wine (Jones, 2018).

This concept of terroir as a “sense of place” has been applied–albeit under different guises—to a wide range of ecological and evolutionary patterns and processes. In ecology, the number of species and their relative abundances at given sites are strongly influenced by local conditions, such as temperature regimes or precipitation schedules (Lembrechts et al., 2019; Meier et al., 2010). In evolutionary biology, the genotypes and phenotypes of populations at different places typically adapt to local conditions because of spatial variation in temperature, precipitation, predators, parasites, or competitors (Endler, 1986; Hereford, 2010; MacColl, 2011; Schluter, 2000). In eco‐evolutionary dynamics, the effects of particular phenotypes and genotypes on ecological processes are highly context‐dependent, varying from place to place in response to local temperatures, nutrients, and moisture levels (Hendry et al., 2017; Johnson & Agrawal, 2005; Tack et al., 2010; Urban et al., 2020). Just as in viticulture, these—and many other—effects of terroir can be seen on very small spatial scales (Kavanagh et al., 2010; Richardson et al., 2014; Richardson & Urban, 2013; Urban et al., 2020; Willi & Hoffmann, 2012).

However, the pure spatial effect of terroir is not always at the fore. As with spatial variation, temporal variation such as interannual temperature or precipitation changes can cause large fluctuations in the abundance of species at any given site (Ash et al., 2017; Ehrlen & Morris, 2015; Van der Putten et al., 2010). Interannual variation in environmental drivers can also act as a selective pressure (Hoffmann & Sgrò, 2011; Siepielski et al., 2017) that can lead to local adaptations (Hendry et al., 2008; Nosil et al., 2018). In eco‐evolutionary dynamics, interannual variation in weather can dramatically alter the importance of phenotypes in population dynamics (Ezard et al., 2009) and other ecological processes (Hendry et al., 2017).

Finally, these two broad categories of effects—space and time—can interact. That is, the spatial effect of terroir can influence how organisms respond to temporal variation in abiotic or biotic conditions. Stated more broadly, the responses of communities, populations, phenotypes, or genotypes to particular changes in precipitation or other environmental factors can depend on other properties of local environments. In ecology, communities in shaded environments are less sensitive to changing temperatures (Clough et al., 2009; Tscharntke et al., 2011). In evolutionary biology, adaptive responses to climate change vary dramatically among populations of a given species (Both & Visser, 2001). In eco‐evolutionary dynamics, the contributions of trait variation to population growth vary among years in ways that differ between populations (Ezard et al., 2009; Hendry et al., 2017).

A series of questions arise when considering the effect of terroir in ecology, evolution, and eco‐evolutionary dynamics such as (1) What is the relative importance of spatial variation (terroir) versus temporal variation (year) in various patterns and processes? (2) More precisely, to what extent does terroir maintain temporally‐consistent differences among sites (i.e., “main effect” of space) as opposed to shaping site‐specific responses over time (i.e., interaction between space and time)? (3) To what extent do these two broad contributions of terroir differ over various spatial or temporal scales? In a scenario of two populations A and B, (1) main differences in traits will remain among populations despite climate variation across years (higher terroir effect), or traits will change along climate variation despite site differences (higher temporal effect), or (2) traits will differ among populations A and B in a site‐specific way that varies based on climate. Finally, (3) spatial and temporal differences in traits between population A and B can increase/decrease depending on their location and how long have they been monitored. Here, we explore these questions by analyzing a 10‐year dataset of environmental features and phenotypic traits in three populations of Darwin's finches. We then compare our results to those from other classic systems in evolutionary biology. We close with a discussion of how the concept of terroir might be useful in helping to reframe and reinvigorate considerations of how temporal and spatial effects contribute to ecology, evolutionary biology, and eco‐evolutionary dynamics.

### 1.1 Darwin's finches

*Terroir* is likely to be very important for Darwin's finches in the Galapágos because different islands, and even different sites within an island, can show dramatic differences in species composition and—for some species—striking variations in morphological traits (Grant & Grant, 1989; Lack, 1947). A major driver of community and trait variation among sites is food resources, especially seed types and sizes (Grant, 1999; Grant & Grant, 2008, 2014; Schluter & Grant, 1984). These differences in food resources result partly from variation in soil and precipitation, which are themselves the result of differences in physical features, such as elevation, direction of prevailing winds, localized clouds, and solar radiation (Trueman & d'Ozouville, 2010). These physical differences are reasonably consistent through time and thus should generate terroir, which we can quantify as the main effect of spatial variation.

At the same time, many studies have emphasized the impact of inter‐annual variation in rainfall, especially due to El Niño or La Niña events, on food availability, which has been observed to cause rapid shifts in finch communities and traits (Grant & Grant, 2002, 2006, 2008). The extent to which these temporal effects are shared across sites can be quantified as the main effect of year and thus contrasted with the main effect of space (as above). Finally, distinct physical features could generate site‐specific responses to interannual variation. For example, sites at higher elevations might be less susceptible to climate fluctuations because prevailing winds push warm, moist air upward, where—even in dry periods—it condenses and falls as rain (Trueman & d'Ozouville, 2010). We can quantify the importance of this second form of terroir as the interaction between space (site) and time (year).

These effects and their relative impacts have not been formally quantified and compared for Darwin's finches because no study to date has quantified and compared both spatial variation (multiple sites) and temporal variation (multiple years) in the same analysis. We do so here by compiling annual environmental and trait data for three populations of the medium‐ground finch (*Geospiza fortis*) across a 10‐year period. We first use Analysis of Variance (ANOVA) to partition the variation in environmental variables (rainfall and vegetation) into the main effect of site, the main effect of year, and the interaction between site and year. We then use univariate and multivariate ANOVAs for a similar partitioning of beak and body trait data. Finally, we use phenotypic trait trajectory analyses (PTA) to explore the contributions of space (site) to temporal changes in multivariate trait means. We conduct these analyses first using all three populations: the small island of Daphne Major and two sites (Academy Bay and El Garrapatero) on the large island of Santa Cruz. Then, because Daphne Major appears to be a special case, we repeat the analyses using only the two sites on Santa Cruz.

## 2 METHODS

### 2.1 Study sites

**FIGURE 1:**
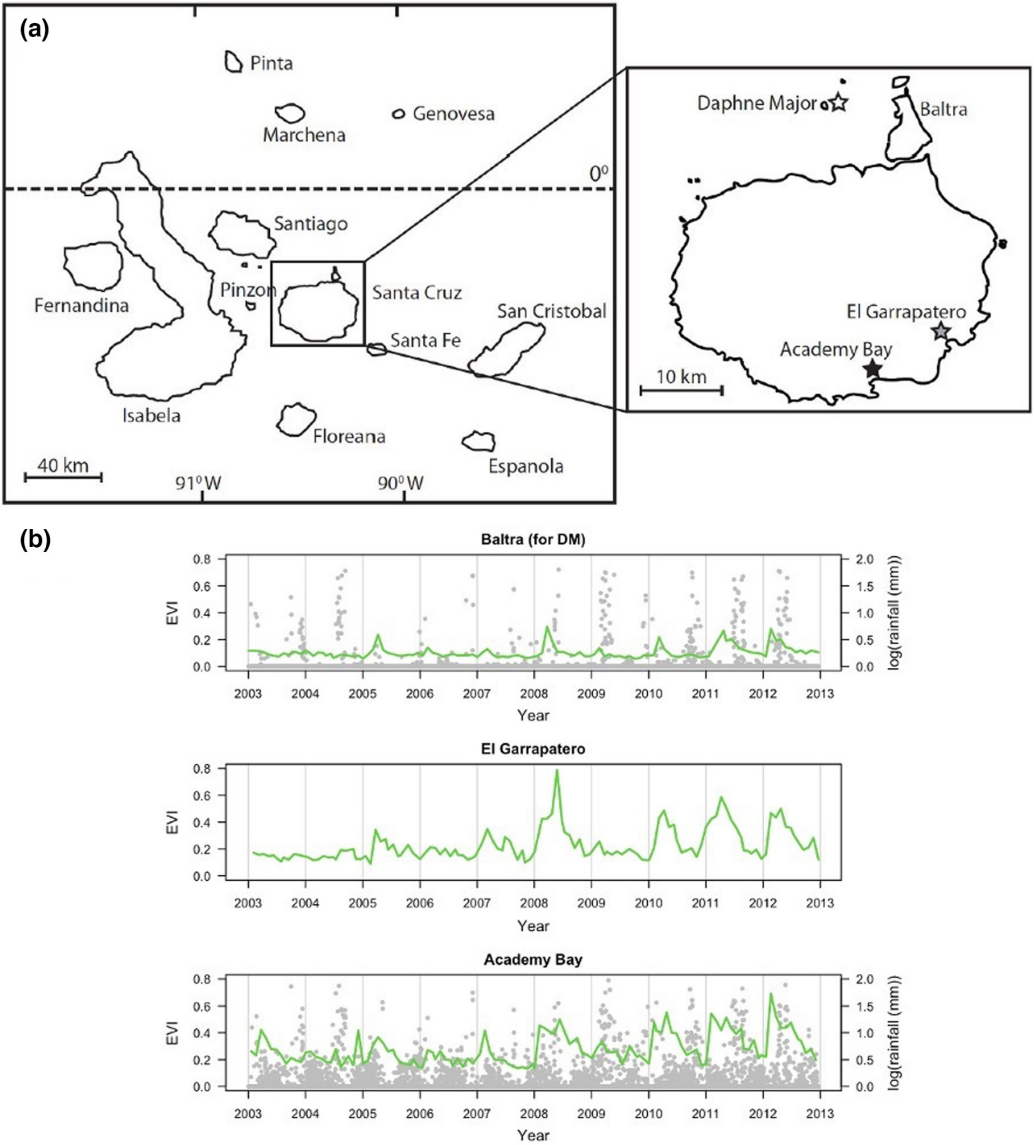
(a) Map of the Galápagos archipelago showing the three study sites: DM for Daphne major (white star), EG for El Garrapatero (gray star), and AB for Academy Bay (black star). (b) Site‐specific daily values for enhanced vegetation index (EVI; green lines) superimposed on daily rainfall (log‐transformed; gray dots) from 2003 to 2012. Rainfall data were not available for El Garrapatero, and values for Daphne major are from the adjacent Baltra Island.

We studied finches from Daphne Major (DM; 0°25′21.1′′S, 90°22′19.6′′ W) and from two lowland sites on the island of Santa Cruz: Academy Bay (AB; 0°44′21.3′′S, 90°18′06.3′′W) and El Garrapatero (EG; 0°41′15.7′′S, 90°13′18.3′′W) (Figure 1a). Academy Bay is located along the southeastern shore of the island, and it is contiguous with the town of Puerto Ayora. El Garrapatero is located along the eastern shore of the island approximately 10 km northeast of Puerto Ayora. El Garrapatero is not adjacent to any human settlement, although a road constructed midway through our sampling regime, in 2008, now passes through our study site, to a parking lot that is used to access a beach (Figure 1a). Daphne Major is located approximately 10 km from the north shore of Santa Cruz (Figure 1a).

### 2.2 Climate and vegetation

We obtained rainfall and spectroradiometric indices of vegetation for the 10 years of our study, 2003–2012 (Figure 1b). Daily rainfall data for Santa Cruz were based on a rain gauge maintained by the Charles Darwin Research Station (Charles Darwin Foundation, 2014). These data are considered representative of both AB (500 m from the gauge) and EG (10 km distant) because the two sites are both on the windward side of the island at similar elevations (20 m for AB, 27 m for EG). However, our personal experience suggests that less rainfall occurs at EG than AB, although no rain gauge was maintained at EG to confirm this suspicion. For DM, we used daily rainfall data from the rain gauge at Baltra Airport, which is 10 km from DM and has a similar climate (Grant & Boag, 1980) and elevation (maximum altitude: 100 m).

Remote sensing data were used to obtain four indices associated with vegetation cover over the 2003–2012 period. More specifically, from the Moderate Resolution Imaging Spectroradiometer (MODIS) database (ORNL DAAC, 2012), we extracted monthly readings for the Normalized Difference Vegetation Index (NDVI), the Enhanced Vegetation Index (EVI), the Leaf Area Index (LAI), and the Fraction of Photosynthetically Active Radiation Index (FPAR) (Figure 1b). These indices are commonly used in studies of spatiotemporal variation in vegetation at global (Alexandridis et al., 2020), regional (Pettorelli et al., 2005), and local (e.g., the Galápagos Islands) (Charney et al., 2021) scales, and they provide robust indicators of primary productivity and vegetation cover state (Charney et al., 2021).

For AB and EG, NDVI and EVI were obtained for an area of 250 m × 250 m (i.e., one pixel), and LAI and FPAR were obtained for an area of 1 km^2^, in each case, the pixel was centered on the sampling area. DM is too small for calculating accurate spectroradiometric indices owing to light reflection from the surrounding ocean. For DM, we therefore used a 250 m × 250 m (for NDVI and EVI) and 1 km^2^ (for LAI and FPAR) area directly north of the Baltra Airport, which is nearby to DM and has similar physical characteristics as explained above.

### 2.3 Capture and measurement of finches

Morphological data were collected for the medium ground finch (*G. fortis*) each year from 2003 to 2012 in the three study sites (DM, AB, EG). In all cases, the birds were captured with mist nets and then banded with uniquely numbered metal leg bands to ensure that individuals were not sampled multiple times. Each bird was inspected and classified—based on plumage, beak color, and the presence of a brood patch—as a juvenile, male, or female (Grant, 1999). Distinguishing females from juveniles sometimes can be difficult, whereas adult males can be readily identified based on their black plumage (Grant, 1999).

Each bird was measured following Boag and Grant (1984, see also Grant, 1999) for beak length (anterior edge of nares to anterior tip of upper mandible), beak depth (at the nares), beak width (at the base of the lower mandible), mass (weight), wing chord (length of longest relaxed right primary feather), and tarsus length (between the nuchal notch at the upper end of the right tarsometatarsus and the lowest undivided scute). Beak and tarsus measurements were made to the nearest 0.01 mm using calipers for EG and AB birds, and dividers (compasses) for DM birds. Wing chord measurements were made to the nearest 0.01 cm using a wing and tail ruler. Mass measurements were made to the nearest 0.01 g using a portable digital scale for AB and EG birds, and a spring scale for DM birds. On DM, each bird was measured by a single person (Peter Grant). At EG and AB, each trait was measured three times (the median value was used for analysis) and measurements were made by multiple people.

### 2.4 Data analyses

#### 2.4.1 Variation in climate and vegetation

Linear fixed‐effect models with Type III Analysis of Variance (ANOVA) were used to examine how spatial variation (main effect of site), temporal variation (main effect of year), and the interaction between these two factors explained variation in rainfall and vegetation indices. Type III Sums of Squares were used given the presence of the site‐by‐year interaction term in our models. The comparisons that could be made were (1) Baltra (for DM) versus AB for log‐transformed daily rainfall, and (2) Baltra (for DM) versus EG versus AB for the monthly average of vegetation indices (EVI, NDVI, FPAR, LAI). Additionally, the same analyses were performed after excluding Baltra (DM) so that we could test the extent of variation between two sites (AB and EG) on the same island. Effect sizes for each of these factors were quantified using partial eta square (*η* ^2^) as suggested in Cohen (1965) when having models with two or more independent variables.

#### 2.4.2 Variation in finch morphology

**FIGURE 2:**
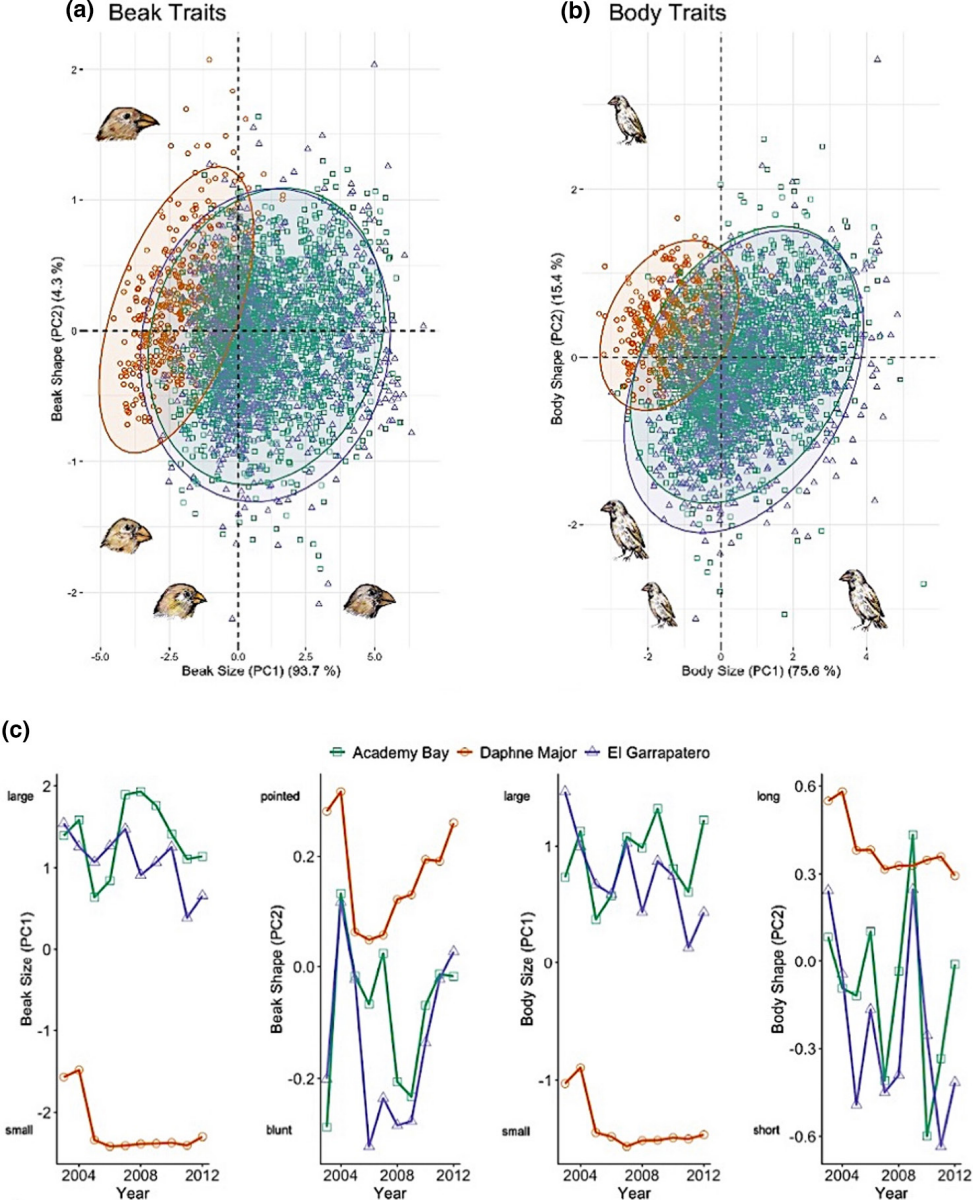
Principal components analysis for (a) beak traits and (b) body traits in *G. fortis* at the three study sites. (c) Trajectories for beak size (PC1), beak shape (PC2), body size (PC1), and body shape (PC2) across 10 years (2003–2012) for the three study sites.

Combining all sites and years, we conducted principal component analyses (PCA) separately for beak traits (length, depth, and width) and then for body traits (mass, tarsus length, and wing chord). PCA based on the covariance matrix was performed for beak traits, following previous analyses (Grant & Grant, 1995), given that all of these traits were measured on the same scale (mm). PCA based on the correlation matrix was used for body traits given the different scales (mm, cm, gr). (Note: the results reported later do not depend on the use of covariance versus correlation matrices.) As in previous work on this species (e.g., Grant, 1999; Grant & Grant, 2002; Hendry et al., 2006, 2009), higher values of PC1 (93.7% of the total variation) correspond to larger beak sizes (positive loadings for all traits) and higher values for PC2 (4.3% of the total variation) correspond to pointier (as opposed to blunter) beaks (positive loadings for beak length and negative loadings for beak depth and beak width) (Figure 2a). For body traits, larger values for PC1 (75.6% of the total variation) correspond to larger bodies overall (positive loadings for all traits), as seen in other work with *G. fortis* (e.g., Grant & Grant, 2006), and larger values for PC2 (15.4% of the total variation) correspond to relatively longer wings (positive loading for wing chord but negative loadings for mass and tarsus length) (Figure 2b).

The resulting values for beak size (PC1 of beak traits), beak shape (PC2 of beak traits), body size (PC1of body traits), and body shape (PC2 of body traits) were analyzed using separate linear fixed‐effect models with Type III ANOVAs applied to quantify the relative contributions (i.e., effect sizes: partial *η* ^2^) of spatial variation (site), temporal variation (year), and their interaction. Sex (male or female) was included in the models as a fixed effect. Juveniles were excluded given that their beak and body traits are still developing (Grant, 1999). Similar to our approach for analyzing climate and vegetation (see above), these finch trait analyses were performed both with and without DM—so as to inform the particular contribution of that small island, and then within‐island site variations, to our assessment of terroir. Finally, all analyses were repeated for adult males only, to test if and how variation in sex ratio might impact our conclusions.

Because the above analyses relied on PCA‐restructured trait (co)variances, as has been typical for research on finches, we also analyzed the original trait measurements in multivariate analyses of variance (MANOVA) using the “Pillai” test, which accounts for our varying sample sizes. These analyses were run separately for beak and body traits, and effect sizes were again quantified as partial *η* ^2^ for the year, site and site‐by‐year interaction terms. As above, we first ran the analyses with the data corresponding to all the study sites (AB, EG, and DM), and then excluding DM.

#### 2.4.3 Variation in phenotypic change trajectories

Phenotypic Trajectory Analysis (PTA: Adams & Collyer, 2009) was used to further explore how terroir (site) might have influenced multivariate trait change across years. For each site, trajectories were generated connecting the multivariate phenotypic means of finch traits at 1 year to the multivariate phenotypic mean of finch traits at the next year. This procedure was done for beak and body traits separately. We then calculated differences between the trajectory lengths (Δ*L*) and directions (angles *θ*) in a pair‐wise fashion (DM vs. EG, AB vs. EG, DM vs. AB). Trajectory length comparisons inform the difference among sites in the amount of among‐year multivariate trait variation along primary axis of interannual change. Trajectory direction comparisons inform the difference among sites in the multivariate orientation of those primary axes of interannual change. See Adams and Collyer (2009) for further explanation of PTA.

#### 2.4.4 Comparison of spatial and temporal effects with other systems

We advocate application of our terroir‐motivated analysis to other patterns in ecology and evolution. We start by placing our findings for *G. fortis* into the context of some other systems that seek to understand the spatiotemporal forces shaping trait variation. To do so, we leveraged studies of multiple populations over multiple years in the ornate tree lizard (*Urosaurus ornatus*: Gilbert & Miles, 2019), the snail kite (*Rostrhamus sociabilis*: Cattau et al., 2018), the Trinidadian guppy (*Poecilia reticulata*: Gotanda & Hendry, 2014), and the pied flycatcher (*Ficedula hypoleuca*: Camacho et al., 2013). In each case, we calculated the variation (partial *η* ^2^) among sites and years from the reported *F*‐values and the degrees of freedom associated to them following Cohen (1965). The resulting partial *η* ^2^ values for each term in each study can then be compared to our own estimates for *G. fortis*.

All the analyses were performed in the statistical program R version 4.1.1 (R Core Team, 2021).

## 3 RESULTS

### 3.1 Climate and vegetation

Yearly averages of the four spectroradiometric indices were strongly correlated with each other at each site (Pearson correlations: all *r* > 0.79; all *p* < .001; *N* = 10 per site), and all of these indices were correlated with total annual rainfall at each site (AB 2003–2012: all *r* > 0.79; all *p* < .0002; *N* = 10; DM 2003–2012: all *r* > 0.73; all *p* < .001; *N* = 10). Not surprisingly, then, all indices yielded similar insights into terroir.

ANOVAs revealed that the strongest effect sizes for rainfall and vegetation cover were associated with site, rather than with year or the site‐by‐year interaction (Table 1). In short, climate and vegetation data suggest very strong and consistent site‐specific environmental differences that should underpin effects of terroir. In particular, DM always had lower rainfall than AB and less vegetation than EG, which in turn always had less vegetation than AB (Figure 1b). This strong and consistent site effect was evident even in the face of dramatic variation across years in overall rainfall across years. In particular, our time series included a dry period from 2003 to 2007 (AB average rainfall = 182.22 mm, Baltra average rainfall = 67.06), followed by a wet period from 2008 to 2012 (AB average rainfall = 536.86 mm, Baltra average rainfall = 284.90) – with the exception of 2009, which was also dry (Figure 1b). This regionally consistent (i.e., across all sites) temporal variation in rainfall was echoed in similarly consistent interannual variation in vegetation cover such that the vegetation indices showed greater values at each site in years where rainfall was greater (Figure 1b). An additional finding from our analyses is that for both rainfall and vegetation, the main effect of year was always stronger than the site‐by‐year interaction. Thus, the primary contribution of terroir was seen in differences among sites that were consistent through time, rather than in a strong contribution of site in modifying the effects of temporal variation.

**TABLE 1:** (a) Total rainfall and average spectroradiometric values for the three study sites from 2003 through 2012. (b) Analysis of variance for log‐transformed rainfall and spectroradiometric values testing for the effect of year, site, and interaction.

(a) Parameter	Daphne major (DM)	El Garrapatero (EG)	Academy Bay (AB)
Rainfall (mm)	148 (±124)	–	360 (±224)
EVI	0.107 (±0.022)	0.227 (±0.071)	0.28 (±0.066)
NDVI	0.245 (±0.043)	0.481 (±0.084)	0.58 (±0.073)
LAI	0.188 (±0.050)	0.579 (±0.189)	1.20 (±0.423)
FPAR	0.098 (±0.024)	0.257 (±0.062)	0.42 (±0.074)

(b) Parameter	Effect	*F*	*p*	*η* ^2^
Rainfall (log)	Year	*F* (9, 7306) = 20.95	**<.0001**	0.02
	Site	*F* (1, 7306) = 258.20	**<.0001**	0.03
	Year * Site	*F* (9, 7306) = 1.36	.1977	0.002
EVI	Year	*F* (9, 549) =23.18	**<.0001**	0.29
	Site	*F* (2, 549) = 223.04	**<.0001**	0.46
	Year * Site	*F* (18, 549) = 3.34	**<.0001**	0.10
NDVI	Year	*F* (9, 549) = 16.77	**<.0001**	0.23
	Site	*F* (2, 549) = 394.53	**<.0001**	0.60
	Year * Site	*F* (18, 549) = 1.95	**.01069**	0.06
LAI	Year	*F* (9, 1289) = 23.62	**<.0001**	0.14
	Site	*F* (2, 1289) = 377.97	**<.0001**	0.38
	Year * Site	*F* (18, 1289) =6.82	**<.0001**	0.09
FPAR	Year	*F* (9, 1289) = 23.03	**<.0001**	0.14
	Site	*F* (2, 1289) = 674.33	**<.0001**	0.52
	Year * Site	*F* (18, 1289) = 3.43	**<.0001**	0.05

*Note*: *p*‐values in bold mark significant effects. *η* ^2^ quantifies effect size. The spectroradiometric data from Baltra Island served as proxy for Daphne major.

Abbreviations: EVI, Enhanced vegetation index; FPAR, Fraction of photosynthetically active radiation; LAI, Leaf area index; NDVI, Normalized difference vegetation index.

After removing DM from the analyses, vegetation index effect sizes decreased (relative to the same term in analyses with DM) by approximately 70% for the main effect of site, increased (relative to the same term in analyses with DM) by approximately 17% for the main effect of year, and decreased by approximately 20% for the site‐by‐year effect. These overall reductions in the relative importance of site suggests that spatial consistency across years across our entire sample is mainly driven by substantial differences between DM and the two Santa Cruz sites (AB and EG). However, it is important to note that site effects were still strong when comparing some vegetation indices within Santa Cruz Island, between EG and AB (Table S1).

### 3.2 Variation in finch morphology

A total of 4388 individuals were captured and measured (AB: 1786, EG: 1229, DM: 1373). PCA‐based analyses showed strikingly smaller beaks, pointier beaks, smaller bodies, and larger wings in *G. fortis* at DM as compared to AB and EG (Figure 2a, b). Further, *G. fortis* at EG and AB were much more variable in all traits than were *G. fortis* at DM (Table 2). These general differences between the finch populations have been reported in earlier analyses that did not simultaneously assess temporal variation (Boag & Grant, 1984; Grant et al., 1985). During our 10‐year study period, mean values for beak and body size typically varied much more among years at AB and EG than at DM (Figure 2c), with exception of an abrupt change between 2004 and 2005 at DM reported as a result of character displacement event reported by Grant and Grant (2006). Beak shape, however, was similarly variable among the three sites (Figure 2c).

**TABLE 2:** Mean and standard error for beak and body traits at the three study sites.

	Beak Traits			Body Traits		
	Beak length (mm)	Beak depth (mm)	Beak width (mm)	Tarsus length (mm)	Wing chord (mm)	Mass (gr)
Academy Bay	11.79 ± 0.022	11.25 ± 0.029	9.93 ± 0.023	20.81 ± 0.031	69.22 ± 0.089	21.35 ± 0.089
Daphne Major	10.46 ± 0.021	8.68 ± 0.020	8.37 ± 0.015	18.99 ± 0.021	66.72 ± 0.062	15.39 ± 0.048
El Garrapatero	11.72 ± 0.028	11.27 ± 0.038	9.91 ± 0.029	21.23 ± 0.038	68.77 ± 0.115	21.35 ± 0.089

**FIGURE 3:**
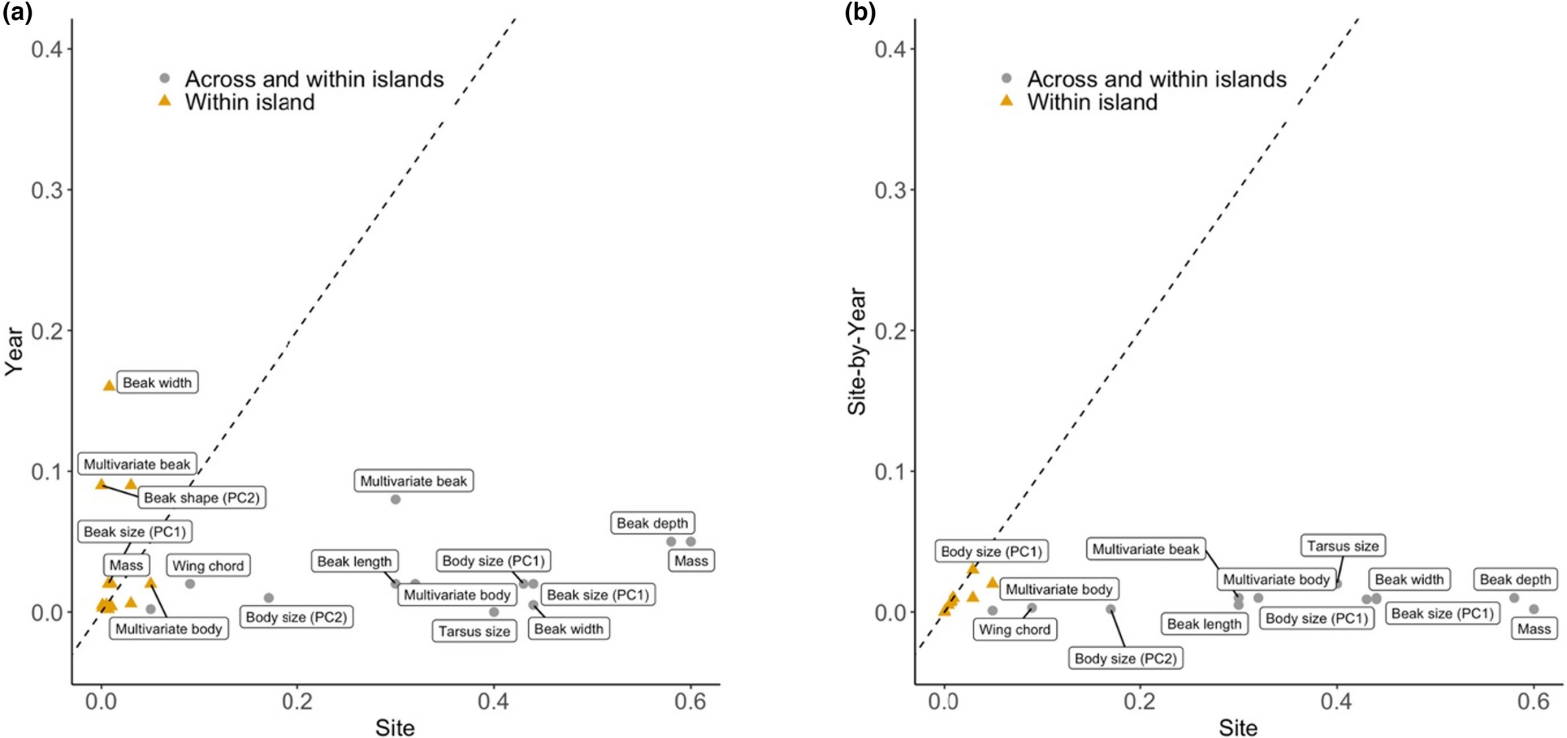
Effect sizes (partial *η* ^2^) for (a) the main effect of site versus the main effect of year, and (b) the main effect of site versus the site‐by‐year interaction for beak length, beak depth, beak width, beak size (PC1), beak shape (PC2), multivariate beak size/shape, mass, tarsus length, wing chord, body size (PC1), body shape (PC2), and multivariate body size/shape for comparisons across islands (gray: AB, DM, EG) and between the two sites on Santa Cruz island (yellow: AB and EG).

Echoing the above‐noted differences between sites, ANOVA and MANOVA analyses indicated that the main effect of site explained most of the variation, followed by the main effect of year and then the site‐by‐year interaction (Table 3; Figure 3). The largest effect sizes for site were evident for beak size (*η* ^2^ = 0.42) and body size (*η* ^2^ = 0.43), both of which were much larger than the corresponding effect for beak shape (*η* ^2^ = 0.05) and body shape (*η* ^2^ = 0.12). The main effect of year and the site‐by‐year interaction were of similar magnitude in all cases (Table 3). That is, interannual variation in *G. fortis* traits had roughly comparable contributions from shared regional changes (main effect of year) and interactions of regional variation with site‐specific factors (site‐by‐year interaction). These results follow those seen for rainfall and vegetation indices in that the main contribution of terroir lies in generating site‐specific phenotypic differences that mainly persist across year.

**TABLE 3:** Analysis of variance (univariate ANOVAs and multivariate MANOVAs) for beak and body traits for *G. fortis* at the three study sites (AB: Academy Bay, EG: El Garrapatero, DM: Daphne major) by year, site, and site‐by‐year interaction including males and females.

	Term	All Populations (AB, EG, DM)			Only AB vs. EG		
		*F*	*p*	*η* ^2^	*F*	*p*	*η* ^2^
Beak Traits							
PC1 (beak size)	Year	*F* (9, 4357) = 6.64	**<.0001**	0.01	*F* (9, 2994) = 4.60	**<.0001**	0.01
	Site	*F* (2, 4357) = 1589.41	**<.0001**	0.42	*F* (1, 2994) = 13.51	**<.0001**	0.005
	Site * Year	*F* (18, 4357) = 4.38	**<.0001**	0.02	*F* (9, 2994) = 4.12	**<.01**	0.01
	Sex	*F* (1, 4357) = 143.75	**<.0001**	0.03	*F* (1, 2994) = 76.29	**<.0001**	0.02
PC2 (beak shape)	Year	*F* (9, 4357) = 13.66	**<.0001**	0.03	*F* (9, 2994) = 16.32	**<.0001**	0.05
	Site	*F* (2, 4357) = 126.42	**<.0001**	0.05	*F* (1, 2994) = 2.81	.093	0.001
	Site * Year	*F* (18, 4357) = 4.44	**<.0001**	0.02	*F* (9, 2994) = 3.05	**<.001**	0.01
	Sex	*F* (1, 4357) = 26.29	**<.0001**	0.006	*F* (1, 2994) = 77.28	**<.0001**	0.03
MANOVA (Beak length, beak depth, beak width)	Year	*F* (9, 4357) = 34.96	**<.0001**	0.07	*F* (9, 2994) = 35.69	**<.0001**	0.09
	Site	*F* (2, 4357) = 642.67	**<.0001**	0.31	*F* (1, 2994) = 19.41	**<.0001**	0.02
	Site * Year	*F* (18, 4357) = 10.28	**<.0001**	0.04	*F* (9, 2994) = 6.20	**<.0001**	0.02
	Sex	*F* (1, 4357) = 68.84	**<.0001**	0.05	*F* (1 2994) = 52.84	**<.0001**	0.05
Body Traits							
PC1 (body size)	Year	*F* (9, 4357) = 10.41	**<.0001**	0.02	*F* (9, 2994) = 7.38	**<.0001**	0.02
	Site	*F* (2, 4357) = 1651.12	**<.0001**	0.43	*F* (1, 2994) = 7.88	**<.0001**	0.003
	Site * Year	*F* (18, 4357) = 4.86	**<.0001**	0.02	*F* (9, 2994) = 4.15	**<.0001**	0.01
	Sex	*F* (1, 4357) = 489.01	**<.0001**	0.10	*F* (1, 2994) = 273.48	**<.0001**	0.08
PC2 (body shape)	Year	*F* (9, 4357) = 29.08	**<.0001**	0.06	*F* (9, 2994) = 22.42	**<.0001**	0.06
	Site	*F* (2, 4357) = 298.19	**<.0001**	0.12	*F* (1, 2994) = 33.58	**<.0001**	0.01
	Site* Year	*F* (18, 4357) = 15.43	**<.0001**	0.06	*F* (9, 2994) = 19.85	**<.0001**	0.06
	Sex	*F* (1, 4357) = 544.88	**<.0001**	0.11	*F* (1, 2994) = 3.33	.0679	0.001
MANOVA (Mass, wing chord, tarsus length)	Year	*F* (9, 4357) = 18.92	**<.0001**	0.04	*F* (9, 2994) = 21.52	**<.0001**	0.06
	Site	*F* (2, 4357) = 694.48	**<.0001**	0.31	*F* (1, 2994) = 42.43	**<.0001**	0.04
	Site * Year	*F* (18, 4357) = 10.61	**<.0001**	0.04	*F* (9,2994) = 8.99	**<.0001**	0.03
	Sex	*F* (1, 4357) = 391.00	**<.0001**	0.21	*F* (1, 29,994) = 291.04	**<.0001**	0.22

*Note*: *p*‐values in bold mark significant differences. Partial eta‐squared (*η* ^2^) quantifies effect size.

After removing DM from ANOVA and MANOVA analyses of finch morphology, overall effect sizes for site decreased (relative to the models with DM) by 95%, year effects increased by 28%, and site‐by‐year effects decreased by 50% (Table 3). Thus, variation was now (considering only AB and EG) explained roughly equally across the year and site‐by‐year terms, which were both slightly greater than the site term. These changes in statistical outcomes reveal that terroir in our *G. fortis* dataset revolves mostly around the beak size and body size (but not shape) of DM birds relative the Santa Cruz (AB and EG) populations.

**FIGURE 4:**
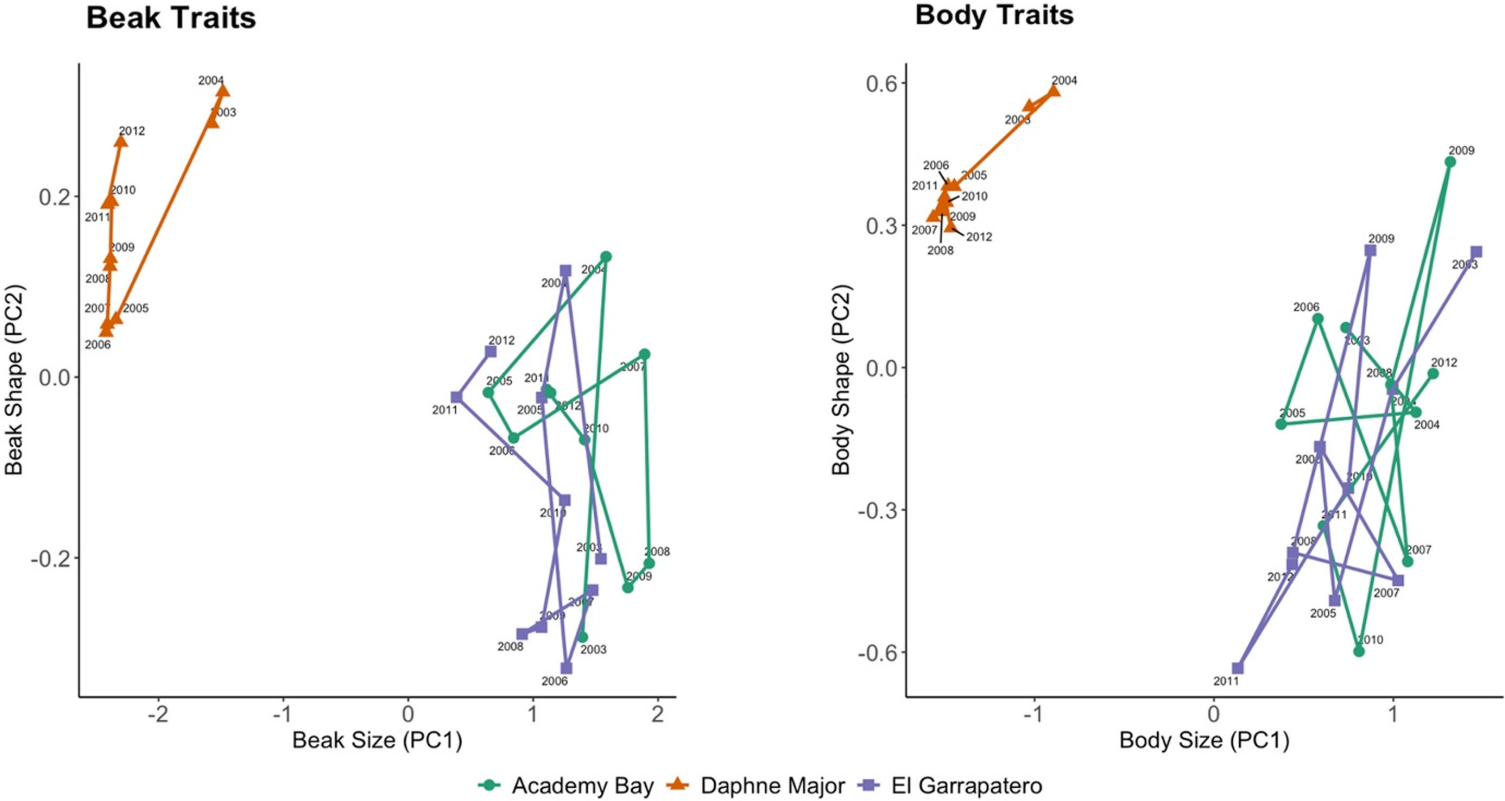
Phenotypic trajectories from phenotypic trajectory analysis across years for beak and body traits at the three study sizes from 2003 to 2012.

Phenotypic trajectory analyses (PTA) revealed differences among sites in the length and direction of the multivariate trajectories for mean beak and body traits (Table 4, Figure 4). That is, the *magnitude* (*ΔL*) and *direction* (θ) of temporal variation in beak and body traits further illustrated the importance of terroir (effect of site) in *G. fortis* traits. Specifically, for beak traits, average differences were greater in the direction of trajectories compared to their magnitude, which indicates the effect of terroir in creating divergent phenotypic trajectories (Table 4). For body traits, terroir equally influenced the differences in magnitude and direction of trajectories (Table 4). When pair‐wise comparisons were made across sites, differences were much larger (and significant) only for DM versus for the other two sites (Table 4). These results again confirm that terroir is mostly driven by the distinctions between DM and the Santa Cruz populations.

**TABLE 4:** (a) Results for phenotypic trajectory analysis (PTA) of *Geospiza fortis* at the three study sites from 2003 to 2012.

	*ΔL* (mm)	*p*‐Value	*θ* (angle degrees)	*p*‐Value
(a)				
Beak traits	1.228	**.005**	119.869	**.001**
Body traits	59.26	**.001**	72.773	**.001**
(b) Population				
Beak traits				
AB vs. DM	2.797	**.002**	21.902	**.006**
AB vs. EG	0.639	.360	6.621	.504
EG vs. DM	2.158	**.005**	27.84	**.001**
Body Traits				
AB vs. DM	13.559	**.001**	28.214	**.001**
AB vs. EG	0.463	.808	18.102	**.028**
EG vs. DM	14.022	**.001**	11.257	.152

*Note*: *ΔL*: Average difference between in the length of trajectories in mm. θ: Average differences in the direction of trajectories given in angle degrees. (b) Pairwise comparisons of phenotypic trajectories between the three study sites (AB: Academy Bay, DM: Daphne major, EG: El Garrapatero). *p*‐values in bold indicate significant differences.

### 3.3 Comparison of spatial and temporal effects with other systems

**FIGURE 5:**
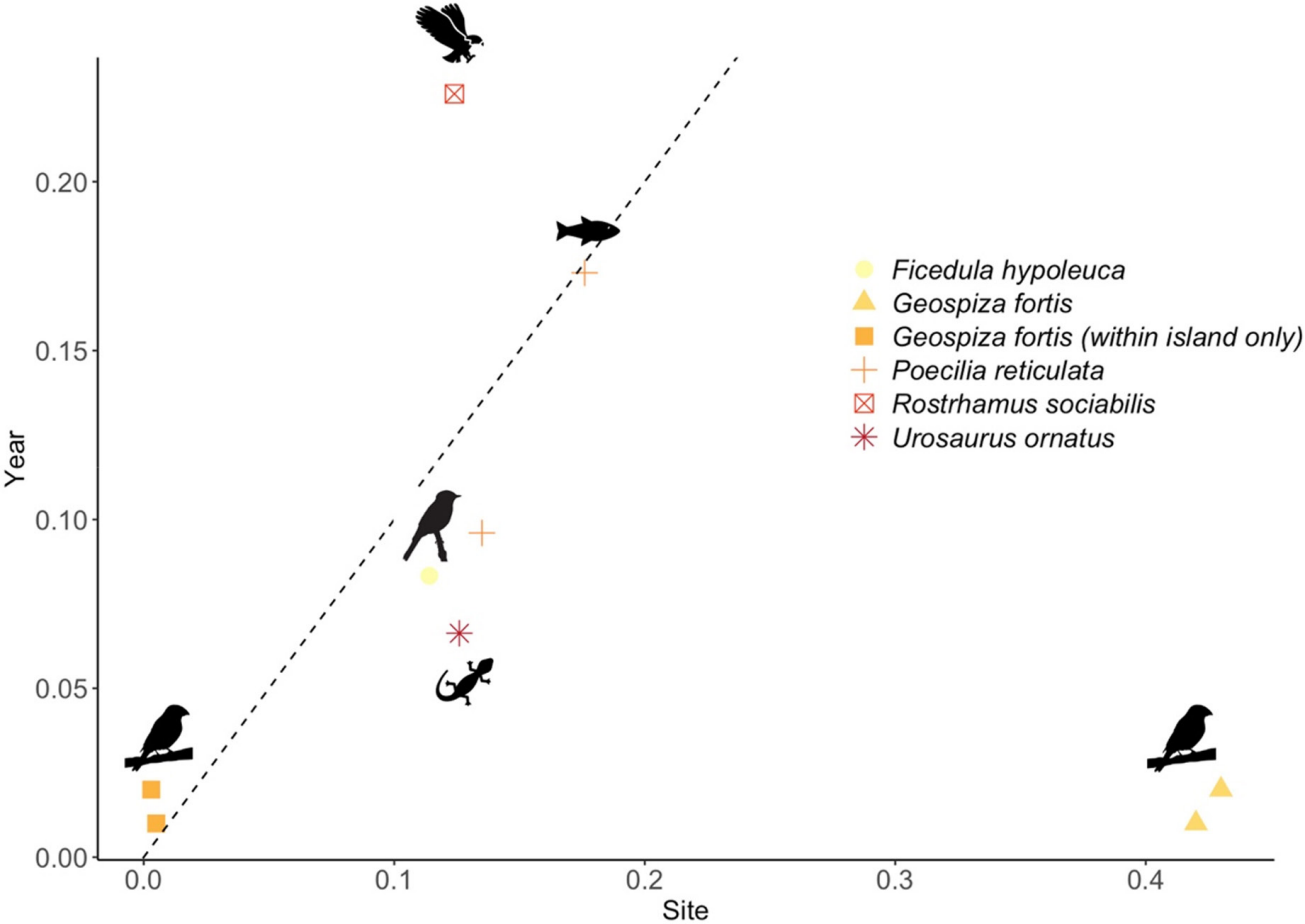
Effect sizes (partial eta‐squared: *η* ^2^) for the main effects of year (temporal) and site (spatial) calculated for different study systems. Each point represents a particular phenotypic trait.

The importance of terroir differed among traits and study systems (Figure 5). Overall, the main effect of site tended to be only slightly greater than the main effect of year across systems, but finch traits showed the highest site effect among all, which suggests that terroir is stronger in finche (when DM is included) than in those other classic systems. When DM was excluded, the main effect of site for finches decreased markedly and was—in fact—lower than the estimates of the other study systems. In short, terroir is exceptionally strong in *G. fortis* in comparison to other systems, but only for the presence of the DM population.

## 4 DISCUSSION

Our use of the term *terroir* is intended to highlight the importance of local biotic and abiotic conditions in shaping organismal attributes. One way that terroir could play out for Darwin's finches would be differences among sites in finch traits and finch community composition. Indeed, spatial differences among finches were the focus of early studies on this group (Bowman, 1961; Grant et al., 1976; Lack, 1947). More recently, however, emphasis has shifted toward temporal changes within finch populations—especially on the island of Daphne Major (Boag & Grant, 1981; Grant & Grant, 2002, 2006; Lamichhaney et al., 2016). At present, the relative importance of these two main factors—that is, spatial and temporal effects—remains unknown for this group—simply because no study has formally assessed both components of variation for a common set of populations over a common time frame.

Our study fills this information gap by analyzing data collected annually over a 10‐year period for three populations of the medium‐ground finch (*G. fortis*). Most prominently, our analysis revealed a very strong signature of “terroir”—that is, temporal changes in beak and body traits were typically small relative to the magnitude of phenotypic differences among sites. Moreover, these patterns of trait variation closely mirrored the strong and temporally consistent differences among sites in climate (rainfall) and vegetation indices (Table 1, Figure 1b). Importantly, however, the effect of terroir was highly variable among traits and sites. In particular, spatial effects were greatest relative to temporal effects for body and beak *size*, as opposed to beak and body *shape*. Further, spatial effects were greatest when including the small island of Daphne Major, as opposed to just the two sites on Santa Cruz Island (AB and EG) (Table 3, Figure 3). These variable contributions of space and time provide a new context to discuss, evaluate, and interpret the terroir of the finch.

### 4.1 Why is terroir so strong for Darwin's finches?

Terroir could manifest as temporally consistent differences among sites (i.e., the main effect of site) or as site‐specific temporal changes (i.e., the interaction between site and year). Our results mainly fall into the first category; that is, consistent differences among sites tend to be more important than site‐specific temporal changes. This outcome likely reflects physical features of the sites that generate consistent differences in rainfall, which generates consistent differences in plants, which generate consistent differences in finch traits.

The starting point for finch terroir is thought to be topographic differences among sites in relation to wind direction and ocean currents (Trueman & d'Ozouville, 2010). In particular, Daphne Major (DM) is only 0.33 km^2^ with a peak elevation of 120 m, and it falls in the rain shadow (given the prevailing winds) of Santa Cruz (Boag & Grant, 1984b; Snell et al., 1996). Santa Cruz, by contrast, is 986 km^2^ and has a maximal elevation of 855 m, which generates considerable rainfall when prevailing winds push moist air to higher and thus colder elevations (Pryet et al., 2012; Snell et al., 1996). Correspondingly, DM experiences less than half the precipitation and has less than half the vegetation cover of our two Santa Cruz sites (Table 1). Not surprisingly, plant communities and seed distributions differ markedly between DM and Santa Cruz (Abbott et al., 1977). Although it is not possible to confidently link specific seed differences to specific beak differences between these populations, it is at least tempting to note that some foods (e.g., *Cordia lutea* seeds) often eaten by large morphs of *G. fortis* on Santa Cruz (e.g., De León et al., 2014) are lacking on DM (Boag & Grant, 1984), where these large *G. fortis* are similarly absent.

The two sites on Santa Cruz—Academy Bay (AB) and El Garrapatero (EG)—are both located in the lowlands and are more similar to each other—in all respects—than either site is to DM. For instance, average values for *G. fortis* traits did not differ consistently between the two sites. Instead, the only noteworthy difference between these populations is in modality of the beak size distribution, with bimodality more evident at EG than at AB (Hendry et al., 2006). We should note that these differences in modality do likely reflect some aspect of terroir. For example, AB has greater vegetation cover than does EG (Table 1), at least in part due to their different positions along the coast of Santa Cruz (southeastern vs. eastern shore). Further, AB has approximately twice the overall seed abundance as does EG (De León et al., 2011). However, the most likely reason for differences in modality is the role of recent human influences. AB (but not EG) is located next to a human settlement. A meta‐analysis performed by Liu and Niyogi (2019) found an average rainfall increase of 16% in sites close to urban settlements, and indeed our own personal experience suggests that rainfall was more frequent and heavier at AB than at EG (no rainfall gauge is present at EG to confirm this experience). Further, AB houses many exotic plants and human foods that are used by finches (De León et al., 2011, 2019). These various human influences at AB appear to break down the diet‐morphology‐performance relationships that are critical to maintain bimodality in *G. fortis* beak size (De León et al., 2011, 2019; Hendry et al., 2006).

A second‐order result of our analysis was that terroir appears to be much more important for beak and body *size* than for beak and body shape, the later mainly being relative wing length (Figure 3). Previous studies have highlighted important differences in beak shape *among* finch species (Bowman, 1961; Foster et al., 2008); however, differences in beak shape *within Geospiza* species are less striking (Foster et al., 2008). Perhaps the main reason is that *G. fortis*—whether large or small—tend to crack seeds in a similar way by exerting bite forces that relate to beak depth and width rather than beak length (Herrel et al., 2005a, 2005b). Beak length, by contrast, seems to be associated with food manipulation (Grant, 1999; Price et al., 1984). Hence, selection on beak size might be strongly divergent (or disruptive), whereas selection on beak shape might be stabilizing for optimal manipulation, irrespective of seed size. Of course, this statement is a speculative generalization given that different food types do, in fact, require different beak movements (Grant, 1981). Further, other forces, such as gene flow, can influence beak shape. For instance, introgression into *G. fortis* from *G. scandens* has led to an increase in beak length of *G. fortis* (Grant & Grant, 2002). In summary, our main point here is not that the effects of terroir are absent for beak shape—merely that they are much weaker than for beak size.

### 4.2 Why is Daphne major special?

Our results indicate that *terroir* makes a very strong contribution to beak and body size variation—but really only due to the inclusion of DM. On average, *G. fortis* at DM have 23% deeper beaks, 17% longer beaks, and 30% lighter bodies than do finches at AB and EG (Table 2, Figure 2). This observation is not a new one, as previous studies have emphasized the relatively small size of DM *G. fortis* and the relatively large size of Santa Cruz *G. fortis* (Boag & Grant, 1984; Brüniche‐Olsen et al., 2019; Grant et al., 1985; McKay & Zink, 2015). Not surprisingly, then, our estimates of the importance of terroir drop dramatically when we remove DM from the analyses (Table 2, Figure 3). To explain the particular importance of terroir for DM birds, we here summarize four possible contributors: overall “harshness,” habitat complexity, competitive interactions, and gene flow/introgression.

First, as previously mentioned, DM is much drier and has less vegetation than AB or EG, a difference verified by our vegetation indices. Hence, smaller body sizes (and thus smaller beak sizes) might reflect their more extreme and challenging environment. This hypothesis could be tested by analyzing phenotypic variation among additional populations in relation to average climate and vegetation measures. *G. fortis* exist on many islands and existing finch data (Grant, 1999; Grant & Grant, 2008; Lack, 1947; Schluter & Grant, 1984) could be combined with newly available remote sensing datasets to achieve this goal. At the same time, overall local climate harshness cannot be the only reason for the distinctiveness of the DM site. For instance, the morphology of *G. fortis* at Borrero Bay on Santa Cruz is more similar to that at climatologically‐ different AB (~26 km away) and EG (~25 km away) (Foster et al., 2008) than to climatologically similar DM (~10 km away) (Grant et al., 1985).

Second, DM offers a much smaller and more homogeneous habitat than does EG or AB, or Santa Cruz as a whole, which supports extremely diverse habitats (Trueman & d'Ozouville, 2010). As a result, Santa Cruz should be able to support a wider diversity of phenotypes within species than would be possible on DM. Indeed, the primary cause of the average beak size difference between *G. fortis* on the two islands is not that Santa Cruz lacks small *G. fortis*, but rather that DM lacks large *G. fortis*: that is, the range of beak sizes is greater on Santa Cruz, especially at the large end of the distribution (Grant & Grant, 2014). It seems likely that the more diverse range of food types on Santa Cruz (Abbott et al., 1977) contributes to a greater range of intraspecific variation, which then shapes persistent differences in average beak size between Santa Cruz and DM.

Third, composition of the finch community on DM differs from that at AB and EG, which could precipitate divergent patterns of selection. For starters, only DM lacks the small ground finch (*Geospiza fuliginosa*), which could favor smaller *G. fortis* individuals who could take advantage of the smaller seeds that *G. fuliginosa* would otherwise eat. Further, the colonization and rapid increase of the large ground finch population (*Geospiza magnirostris*) on DM precipitated a character‐displacement shift toward even smaller beak sizes (Grant & Grant, 2006). Thus, it seems possible that different patterns of interspecific competition contribute to why *G. fortis* on DM are so much smaller (on average) than those on Santa Cruz.

Fourth, divergence of finch traits between DM and Santa Cruz could be driven by distinct patterns of gene flow from other *G. fortis* populations or other *Geospiza* species. In particular, hybridization between *G. fortis* and *G. magnirostris* on Santa Cruz might have seeded the genetic variation necessary for the evolution of large *G. fortis* there (Chaves et al., 2016). By contrast, *G. magnirostris* has colonized DM only recently (Gibbs & Grant, 1987; Grant & Grant, 1995), which would limit the scope for gene flow effects. Further, gene flow appears to be substantial for *G. fortis* across Santa Cruz, with only minimal genetic differences over even large distances (De León et al., 2010). By contrast, *G. fortis* immigrants to DM are relatively rare (Grant & Grant, 2009, 2010). Hence, *G. fortis* on DM might—by virtue of their spatial isolation—have more ability to independently evolve to local optima.

In summary, the distinctive nature of the DM *G. fortis* terroir probably reflects a combination of environmental differences and isolation that together shape ecological and evolutionary responses to local conditions. That is, differences in terroir are much more likely to cause differences in communities and traits when places with different properties are not linked by the movement of materials or organisms. This view comports with the classic interpretation of beak traits in finches being shaped by the combination of local food resources (Schluter & Grant, 1984), interspecific competition (Grant & Grant, 2006; Schluter, 2000; Schluter & Grant, 1984), and patterns of gene flow or introgression (Chaves et al., 2016; Farrington et al., 2014; Grant & Grant, 2009, 2010; Petren et al., 2005).

### 4.3 Are Darwin's finches special compared to other systems?

Despite the site effect in finches being the largest among systems due to the presence of the DM population. It is important to note, however, that our two Santa Cruz sites (EG and AB) were in similar lowland arid habitats, whereas *G. fortis* in other habitats on Santa Cruz and on other islands might also show a stronger signal of terroir. Indeed, work on another ground finch species *G. fuliginosa* has reported noteworthy beak and foot size differences between vegetation and climatic zones on Santa Cruz (Kleindorfer et al., 2006). Future work would benefit from adding more diverse habitats on Santa Cruz, thus helping to separate the classic driver of terroir (environmental conditions) from the importance of isolation (DM).

Finally, we note that the small interannual effects (relative to site effects) in our study system could be due to the fact that Darwin's finches are long lived, and that beak size is very strongly genetically determined (Chaves et al., 2016; Lamichhaney et al., 2016). Hence, a 10‐year period might be insufficient to observe dramatic evolutionary changes similar to those found among sites. However, organisms that have short generation times (e.g., guppies) also often show stronger spatial than temporal variation (Figure 5; Gotanda & Hendry, 2014). Further, studies have shown how evolutionary changes in Darwin's finches can happen over only a few years (Grant & Grant, 2002; Lamichhaney et al., 2016). Longer monitoring during more consistent changes in climate (e.g., due to global warming) could perhaps resolve these uncertainties.

## 5 CONCLUSIONS

The large effect of site or “terroir” in explaining not only the phenotypic variation in finches but also the environmental characteristics associated with food availability reinforce the classic hypothesis that diversification in Darwin's finches is driven by ecological differences among locations (Bowman, 1961; Grant, 1999; Lack, 1947; Schluter & Grant, 1984). This realization brings some needed perspective to the current emphasis on contemporary evolution of beak size within finch populations (e.g., Chaves et al., 2016; Lamichhaney et al., 2016). That is, recent studies have highlighted the influence of temporal changes in beak traits by prolonged droughts caused by La Niña or abundant rains caused by El Niño (Grant & Grant, 2002, 2006). Yet, our results make clear that such contemporary or “rapid” evolution within a population is very small relative to spatial factors that have generated consistent spatial variation—and thus driven the radiation of Darwin's finches. Perhaps evolution is extremely rapid when finches colonize a new environment; but, after that, it wobbles around much more subtly around a local optimal dictated by temporally consistent environmental variation. Our results lay the groundwork for further studies that include other islands and sites with different conditions for Darwin's finches. Further, we encourage exploration of the spatio‐temporal evolutionary variation of species with different life histories.

## AUTHOR CONTRIBUTIONS

**Paola Carrion‐Avilés:** Data curation (lead); formal analysis (lead); investigation (equal); methodology (equal); software (equal); validation (equal); visualization (equal); writing – original draft (lead); writing – review and editing (lead). **Joost Raeymaekers:** Conceptualization (lead); data curation (equal); formal analysis (equal); investigation (equal); methodology (equal); project administration (lead); validation (equal); writing – original draft (equal); writing – review and editing (equal). **Luis F. De León:** Investigation (equal); methodology (supporting); writing – review and editing (equal). **Jaime Chaves:** Investigation (equal); methodology (equal); writing – review and editing (equal). **Diana Sharpe:** Investigation (equal); methodology (equal); writing – review and editing (equal). **Sarah Huber:** Investigation (equal); methodology (equal); writing – review and editing (equal). **Anthony Herrel:** Investigation (equal); methodology (equal); writing – review and editing (equal). **Bieke Vanhooydonck:** Investigation (equal); methodology (equal); writing – review and editing (equal). **Kiyoko Gotanda:** Formal analysis (supporting); investigation (equal); methodology (equal); visualization (supporting); writing – review and editing (equal). **Jennifer Koop:** Investigation (equal); methodology (equal); writing – review and editing (equal). **Sarah A. Knutie:** Investigation (equal); methodology (equal); writing – review and editing (equal). **Dale Clayton:** Investigation (equal); methodology (equal); writing – review and editing (equal). **Jeff Podos:** Investigation (equal); methodology (equal); writing – review and editing (equal). **Andrew Hendry:** Conceptualization (lead); funding acquisition (lead); investigation (equal); methodology (equal); project administration (lead); supervision (lead); validation (lead); writing – original draft (equal); writing – review and editing (lead).

## CONFLICT OF INTEREST

The authors declare no conflicts of interest.

## Supporting information


Tables S1‐S2

## Data Availability

The morphological data are available on Dryad for Daphne Major (https://doi.org/10.5061/dryad.g6g3h), and on OSF for Santa Cruz island (https://doi.org/10.17605/OSF.IO/5AFUS). Environmental data and scripts of statistical analyses are available on OSF (https://doi.org/10.17605/OSF.IO/5AFUS).
