# Supplementary material for: The terroir of the finch: How spatial and temporal variation shapes phenotypic traits in DARWIN'S finches
Source: Ecol Evol. 2022 Oct 5;12(10):e9399. doi: 10.1002/ece3.9399 (PMC9534727; doi:10.1002/ece3.9399)
Supplement: Supplementary file 1 — Tables S1‐S2 [file ECE3-12-e9399-s001.docx]

APPENDIX

Table S1. Analysis of variance for spectroradiometric values testing for the effect of year, site, and interaction between Academy Bay and El Garrapatero, exclusively. P-values in bold mark significant effects. η2 quantifies effect size. EVI: enhanced vegetation index; NDVI: normalized difference vegetation index; LAI: Leaf Area Index; FPAR: Fraction of Photosynthetically Active Radiation.

| Parameter | Effect | F | *p* | η2 |
| --- | --- | --- | --- | --- |
| EVI | Year | F (9,359) = 19.82 | **< 0.0001** | 0.34 |
|  | Site | F (1, 359) = 25.89 | **< 0.001** | 0.07 |
|  | Year * Site | F (9, 359) = 1.53 | 0.1344 | 0.04 |
| NDVI | Year | F (9,359) = 12.84 | **< 0.0001** | 0.25 |
|  | Site | F (1,359) = 48.65 | **< 0.0001** | 0.13 |
|  | Year * Site | F (9,359) = 2.06 | **0.03228** | 0.05 |
| LAI | Year | F (9,831) = 19.74 | **< 0.0001** | 0.18 |
|  | Site | F (1,831) = 176.55 | **< 0.0001** | 0.18 |
|  | Year * Site | F (9,831) = 4.01 | **< 0.001** | 0.04 |
| FPAR | Year | F (9,831) = 17.50 | **< 0.0001** | 0.16 |
|  | Site | F (1,831) = 217.65 | **< 0.0001** | 0.21 |
|  | Year * Site | F (9,831) = 1.40 | 0.1808 | 0.02 |

Table S2. Analysis of variance (univariate ANOVAs and multivariate MANOVAs) for beak and body traits for *G. fortis* at the three study sites (AB: Academy Bay, EG: El Garrapatero, DM: Daphne Major) by year, site, and site-by-year interaction including MALES only. P-values in bold mark significant differences. Partial eta-squared (η^2^) quantifies effect size.

|  |  | ALL POPULATIONS (AB, EG, DM) | | |  | ONLY AB vs. EG | | |
| --- | --- | --- | --- | --- | --- | --- | --- | --- |
| BEAK TRAITS | Term | F | *p* | η2 |  | F | *p* | η2 |
| PC1 (beak size) | Year | F (9, 2291) = 3.69 | **< 0.0001** | 0.01 |  | F (9, 1399) =2.09 | **< 0.01** | 0.01 |
|  | Site | F (2, 2291) = 877.31 | **< 0.0001** | 0.43 |  | F (1, 1399) = 3.84 | **0.0501** | 0.003 |
|  | Year * Site | F (18, 2291) = 3.71 | **< 0.0001** | 0.03 |  | F (9, 1399) =2.00 | **< 0.01** | 0.01 |
| PC2 (beak shape) | Year | F (9, 2291) = 10.92 | **< 0.0001** | 0.04 |  | F (9, 1399) = 11.20 | **< 0.001** | 0.07 |
|  | Site | F (2, 2291) = 117.76 | **< 0.0001** | 0.09 |  | F (1, 1399) = 2.42 | 0.119 | 0.002 |
|  | Year * Site | F (18, 2291) = 3.39 | **< 0.0001** | 0.03 |  | F (9, 1399) = 2.18 | **< 0.01** | 0.01 |
| Multi-trait | Year | F (9, 2291) = 17.76 | **< 0.0001** | 0.07 |  | F (9,1399) =17.64 | **< 0.0001** | 0.10 |
| (Beak length, beak depth, | Site | F (2, 2291) = 364.11 | **< 0.0001** | 0.32 |  | F (1,1399) 10.97 | **< 0.0001** | 0.02 |
| beak width) | Year * Site | F (18, 2291) = 7.01 | **< 0.0001** | 0.05 |  | F (9,1399) = 3.85 | **< 0.0001** | 0.02 |
|  |  |  |  |  |  |  |  |  |
| BODY TRAITS | Term | F | *p* | η2 |  | F | p | η2 |
| PC1 (body size) | Year | F (9, 2291) = 7.51 | **< 0.0001** | 0.03 |  | F (9, 1399) = 3.79 | **< 0.001** | 0.02 |
|  | Site | F (2, 2291) = 958.90 | **< 0.0001** | 0.46 |  | F (1, 1399) = 3.25 | 0.0713 | 0.002 |
|  | Year * Site | F (18, 2291) = 3.57 | **< 0.0001** | 0.03 |  | F (9, 1399) = 2.00 | **< 0.01** | 0.01 |
| PC2 (body shape) | Year | F (9, 2291) = 13.36 | **< 0.0001** | 0.05 |  | F (9, 1399) = 9.18 | **< 0.0001** | 0.06 |
|  | Site | F (2, 2291) =109.47 | **< 0.0001** | 0.09 |  | F (1, 1399) = 13.78 | **< 0.0001** | 0.01 |
|  | Year * Site | F (18, 2291) = 9.11 | **< 0.0001** | 0.07 |  | F (9, 1399) = 10.65 | **< 0.0001** | 0.06 |
| Multi-trait | Year | F (9, 2291) =8.09 | **< 0.0001** | 0.03 |  | F (9, 1399) = 8.41 | **< 0.0001** | 0.05 |
| (Mass, wing chord, | Site | F (2, 2291) = 363.84 | **< 0.0001** | 0.32 |  | F (1, 1399) = 22.58 | **< 0.0001** | 0.05 |
| tarsus length) | Year * Site | F (18, 2291) = 6.30 | **< 0.0001** | 0.04 |  | F (9, 1399) = 5.46 | **< 0.0001** | 0.03 |
